# Spatial variation characteristics of vegetation phenology and its influencing factors in the subtropical monsoon climate region of southern China

**DOI:** 10.1371/journal.pone.0250825

**Published:** 2021-04-28

**Authors:** Huanhua Peng, Haonan Xia, Hao Chen, Panding Zhi, Zhonglin Xu

**Affiliations:** 1 National-Local Joint Engineering Laboratory of Geo-Spatial Information Technology, Hunan University of Science and Technology, Xiangtan, China; 2 Hunan Provincial Key Laboratory of Geo-information Engineering in Surveying, Mapping and Remote Sensing, Hunan University of Science and Technology, Xiangtan, China; 3 College of Resources and Environmental Sciences, Xinjiang University, Urumqi, China; Chinese Academy of Sciences, CHINA

## Abstract

Understanding the response mechanism of ecosystems to climate change and human disturbance can be improved by analyzing the spatial patterns of vegetation phenology and its influencing factors. Because the diverse phenological patterns are impacted by cloud cover contamination issues in the satellite observations, there are few remote sensing phenological research data in subtropical monsoon climate regions. To better understand the horizontal and vertical changes of vegetation phenology in these regions and how it may be affected by climatic factors and topographical features, we first extracted vegetation phenological information (such as start of growth season (SOS), end of growth season (EOS) and length of growth season (LEN)) from a reconstructed MODIS EVI time-series data. We then used geographic detectors to identify the influencing factors of phenology in different elevation zoning areas. We have found that in the Xiangjiang River Basin: 1) gradual changes in the longitudinal or latitudinal gradient of vegetation phenology were not obvious. Instead of horizontal changes, the variation pattern of phenology was similar to the striped river network of the Xiangjiang River. Earlier SOS mainly appeared in the areas far away from the river; later SOS appeared in the midstream and downstream reaches.2) Elevation played an important role in the regional differentiation of phenology. Boundaries at elevations of 320 m and 520 m distinctly separated the region into plain, hilly, and mountain vegetation phenological characteristics. 3) The impacts of climatic factors were quite different in the three vertical zoning areas. Precipitation was the most crucial factor affecting SOS both in plain and mountain areas. There was no significant factor affecting EOS in the plain area, but temperature had an essential effect on EOS in the mountain area. The hilly areas had a concentrated growth period with no significant factors affecting phenology. These findings highlight the importance of elevation in phenology at a watershed scale, enhance our understanding of the impact of climate changes on subtropical ecosystems, and provide a reference for further land-use change monitoring.

## Introduction

Vegetation is an important component of terrestrial ecosystems and plays an essential role in the global biogeochemical and energy cycles [1, 2]. The phenology of vegetation is the most sensitive indicator of seasonal changes in environmental conditions [3–6] and reflects the rapid response of vegetation to climate and environmental changes [4, 7, 8]. With the intensification of global climate change, the study of vegetation phenology has attracted wide attention. Numerous researchers have collected long-term records of vegetation phenology by employing fixed-point ground observations [9, 10], phenology camera network [11, 12], and aerospace remote sensing monitoring [6, 13, 14]. In particular, remote sensing has become an important technical means of phenology observation by offering global coverage data for large-scale phenological research [8, 15–17]. The application of remote sensing technology to phenological monitoring mainly includes the characteristics of vegetation phenological change in regional terrestrial ecosystems [14, 18] and its response to climate change and human disturbance [19, 20]; land cover classification and monitoring [21, 22]; and crop phenology monitoring and crop yield estimation [15, 23–25].

Given the importance of phenology to terrestrial ecosystems and recent climate changes, much attention has been focused on climate-sensitive regions, such as spatially heterogeneous semi-arid or arid ecosystems [26–28], tropical forests with a challenging and debated seasonal cycle [29, 30], or high latitudes experiencing rapid warming [8, 20] because these regions have more extensive vegetation phenological changes. The feedback of vegetation to climate changes is stronger in these climate-sensitive areas [14, 26, 31]. For example, in most high-altitude ecosystems, long-term records of phenology suggest that vegetation phenology can be influenced by temperature and precipitation sensitivity [8, 20, 32, 33]. However, to the best of our knowledge, much less attention has been devoted to subtropical climate areas [16, 34, 35], which have abundant biodiversity, complex stand structures, and diverse phenological patterns. The present challenge in capturing phenological dynamics from remote sensing is that vegetation often grows near its thermal optimum, resulting in a weakening of the sensitivity of phenology to climate changes in subtropical areas [17, 36]. Moreover, cloud cover contamination issues of the satellite observations may further reduce the number of optimal data available to represent the phenology interval in this area [13]. Climatic factors, however, are not invariable across a range of conditions. Phenology in different climatic regions suggests that temperature or precipitation sensitivity can vary in space and time [37, 38]. Consequently, phenology research based on remote sensing needs to be thorough to assess whether vegetation phenology changes in subtropical monsoon climate regions are consistent with those observed in climate-sensitive areas.

In addition to climatic factors, topographical features also control the distribution of vegetation and have a greater impact on phenology [28, 39]. On the global scale, changes in vegetation homogenization along elevational gradients can be divided into different modes. In southeast China, these changes mainly manifested in increased vegetation homogenization [39]. It is generally believed that phenology is affected by temperature variability at high altitudes, while it is more strongly limited by water stress in lowlands [40]. However, the mechanisms governing the spatial dynamics of the elevational gradients of phenology are not universal. Du *et al*. [28] observed that the negative correlation between green-up dates and precipitation gradually weakened or even switched to a positive one over the elevational gradients in the Qilian Mountains. There are also opposite trends in phenology changes at different elevational gradients due to the topography having remarkable effects on temperature, precipitation, and humidity [34]. The phenological changes caused by topography are pronounced at large regional scales. In contrast, at the watershed scale, there are more interactive factors that need to be considered and further refined and verified.

In this study, the Moderate Resolution Imaging Spectroradiometer (MODIS) time-series of data with daily temporal and 250-500m spatial resolution, which has been widely used and tested for phenology retrieval across diverse vegetation and climatic regions [6, 25, 28, 29], was used to extract vegetation phenology in subtropical monsoon region. The objective of this paper is mainly to explore the horizontal and vertical distributions of vegetation phenology in typical subtropical ecosystems of southern China and how they relate to climate, topography, and other factors. The primary contents of this study are: 1) to analyze the spatial patterns of vegetation phenology at the watershed scale, 2) to explore the horizontal and vertical distributions of the SOS, EOS, and LEN, and 3) to reveal the correlation between phenology, climate factors, and topographic factors. We hope this research will provide the basis for monitoring vegetation phenological change at the watershed scale, and provide meaningful guidance for analyzing the response of subtropical vegetation phenology to climate change.

## Materials and methods

### Study area

**Fig 1:**
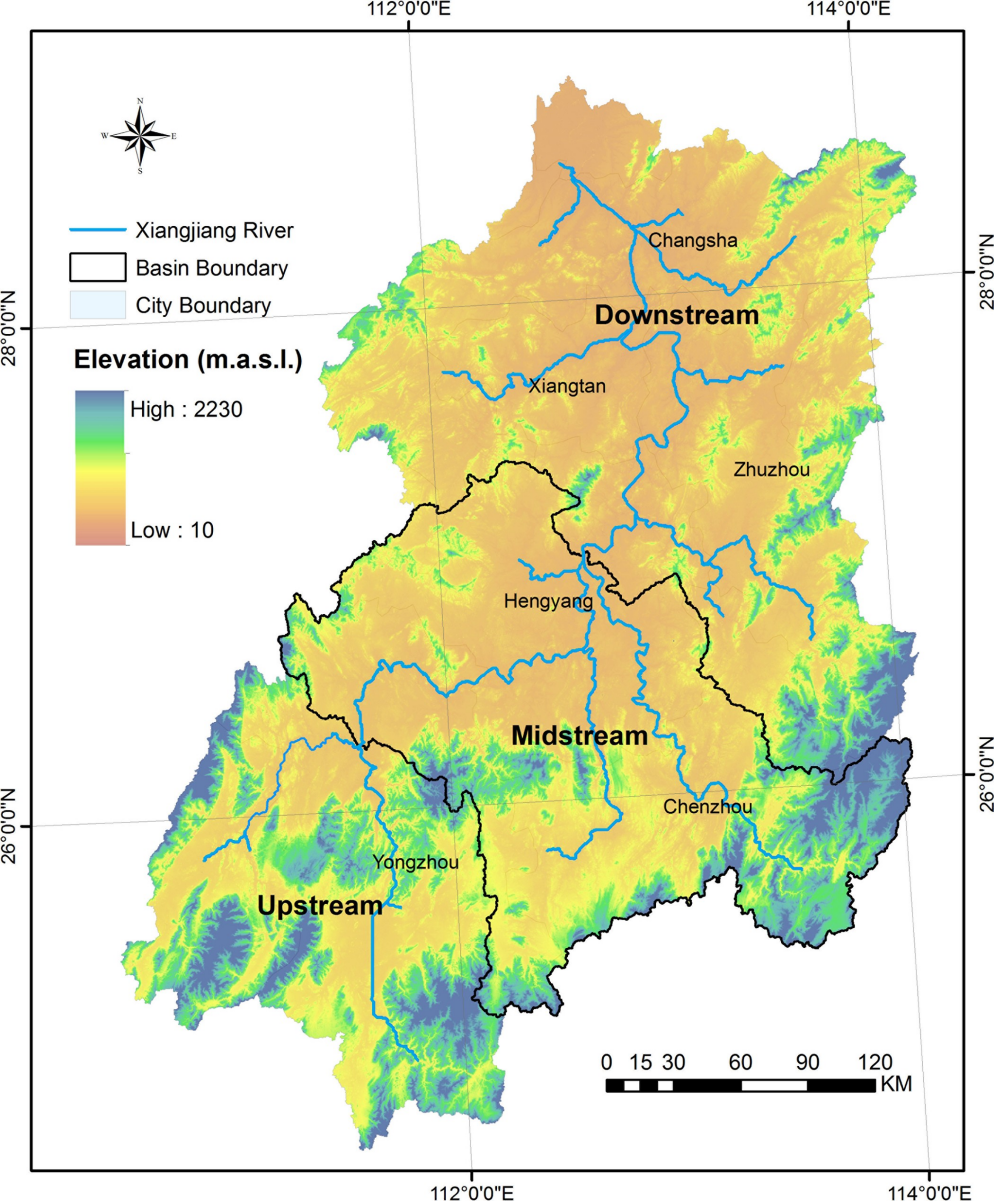
Digital elevation model of the Xiangjiang River basin and its watershed characteristics. (Source of base map was provided by Shuhan Wang. The map was generated by using the free and open source software QGIS version 3.16 (http://www.qgis.org/en/site/)).

The Xiangjiang River Basin, with a total area of 94 660 km^2^, is located between 24°35′ - 28°50′ N and 110°30′ - 114°20′ E. It belongs to the typical subtropical monsoon humid climate area of southern China. The average annual temperature of the basin is 16–18°C, and the average annual precipitation is 1300–1700 mm. The rainy season coincides with the hotter period. This basin is surrounded by mountains on three sides, with low topography in the middle region. The upper reaches of the Xiangjiang River flow through mountain areas, the midstream flows through low-mountain and hilly areas, while the downstream has low hilly-plain topography (Fig 1). The southern part of the basin is mainly covered by evergreen broad-leaved forests, coniferous forests, and pine forests. Agricultural planting, herbs, and cultivated forests are the main vegetation types in the middle and lower basin. The compositions of the vegetation community are typical types for the development of vegetation phenological in subtropical ecosystems.

### Data collection

In this paper, information on vegetation phenology was derived from the 16-day 250-meter spatial resolution MODIS vegetation index product (MOD13Q1). The dataset was downloaded from the National Aeronautics and Space Administration (NASA) Land Processes Distributed Active Archive Center (LPDAAC) Working Group website for the period January 2001 to December 2015. The MOD13Q1 dataset mainly contains 16-day Normalized Indices and Vegetation Index (NDVI) and Enhanced Vegetation Index (EVI). Compared with NDVI, the EVI uses the Constrained View Angle Maximum Value Composite (CV-MVC) and the Bidirectional Reflectance Distribution Function (BRDF)/CV-MVC to avoid the saturation problem of ratio-based vegetation indices [41]. Therefore, in this study, the EVI vegetation index in the 16-day synthetic product of MODIS was used to retrieve the timing of vegetation growth. The MODIS Reprojection Tool (MRT) was used to convert, extract, re-sample, mosaic, and re-project the MOD13Q1 EVI data [42].

We used the monthly average dataset at 1 km grid spatial interpolation of temperature and precipitation for China from 2001 to 2015. The data was obtained from the National Earth System Science Data Center, National Science & Technology Infrastructure of China (http://www.geodata.cn). The ASTER GDEM V2 data, downloaded from the USGS website (https://earthexplorer.usgs.gov), was used to extract the elevation of the watershed. Based on the spatial resolution of the MODIS EVI data, temperature, precipitation, and elevation data were re-sampled to 250 m by median filtering and nearest neighbor re-sampling methods.

### Phenology extraction

Although the EVI algorithm has been devised to improve the quality of the original remote sensing images in the production process of MOD13Q1 data, the EVI data still contains clouds, aerosols, and other noise, which may lead to the detection of false changes in vegetation. Therefore, it is necessary to pre-process the EVI data. The Savitzky-Golay filter, a curve fitting method, was used to reconstruct the EVI time series vegetation index data set [43]:

$$Y_{j}^{*}=\sum_{i=-m}^{i=m}\frac{C_{i}Y_{j+i}}{N}$$ (1)

where, *Y*_j_^*^ is the newly synthesized sequence data, *Y*_*(j+i)*_ represents the original sequence data, *C*_i_ is the filtering coefficient, and *N* is the data point included in the sliding window. In this paper, we set *C*_*i*_ by 2.0 adaptive strength, no peak filtering, 0.5 seasonal parameters, and 2 Savitzky-Golay windows.

From a remote sensing perspective, vegetation phenological metrics are typically associated with annual vegetation changes interpretable from remote sensing images [44]. In this study, the start of growth season (SOS), end of growth season (EOS), and length of growth season (LEN) were selected as phenological indicators of vegetation growth. The TIMESAT software package [45] was used to extract the maximum and minimum values of the yearly reconstructed EVI time series data, and a dynamic threshold method was used to determine the annual difference to extract the beginning and end stages of phenology [46]. SOS was defined as the time point when EVI exceeded 20% of the current EVI amplitude (the difference between the maximum and the minimum), while the time point at which EVI decreased to 20% of the current EVI amplitude was identified as EOS.

### Analytical approaches

Correlation analysis is a direct and effective approach to reveal the impact of climate factors on phenology [8, 14, 47]. The Spearman correlation coefficients between the phenological indicators and monthly temperature/precipitation were computed over the study period. Furthermore, the geographic detector, a typical method widely used to reveal the spatial correlation between two variables [48], was applied to explore the relative importance of phenology and its influencing factors. The basic idea of this method is that if the spatial pattern of an independent variable(e.g., temperature) is similar to that of a dependent variable(e.g., SOS), this variable will have an important influence on the dependent variable [49]. The power of determinant (q) can be measured by the following formula:

$$q=1-\frac{\sum_{h=1}^{L}N_{h}\sigma_{h}^{2}}{N\sigma^{2}}$$ (2)

where *L* is the number of strata of the potential factors, and *N*_*h*_ and *N* are the numbers of grids of the strata *h* and the whole basin, respectively. The $\sigma_{h}^{2}$ and *σ*^2^ are the variances of each potential factor and the vegetation phenological indicators, respectively. The value range of q is [0, 1], and the greater the value of q, the greater the influence of the factor is. In extreme cases, q = 1 indicates that the spatial pattern of vegetation phenological changes is entirely determined by the influencing factors, while q = 0 means no association between the vegetation phenological indicators and the influential factors.

## Results and analysis

### Spatial patterns of phenology

**Fig 2:**
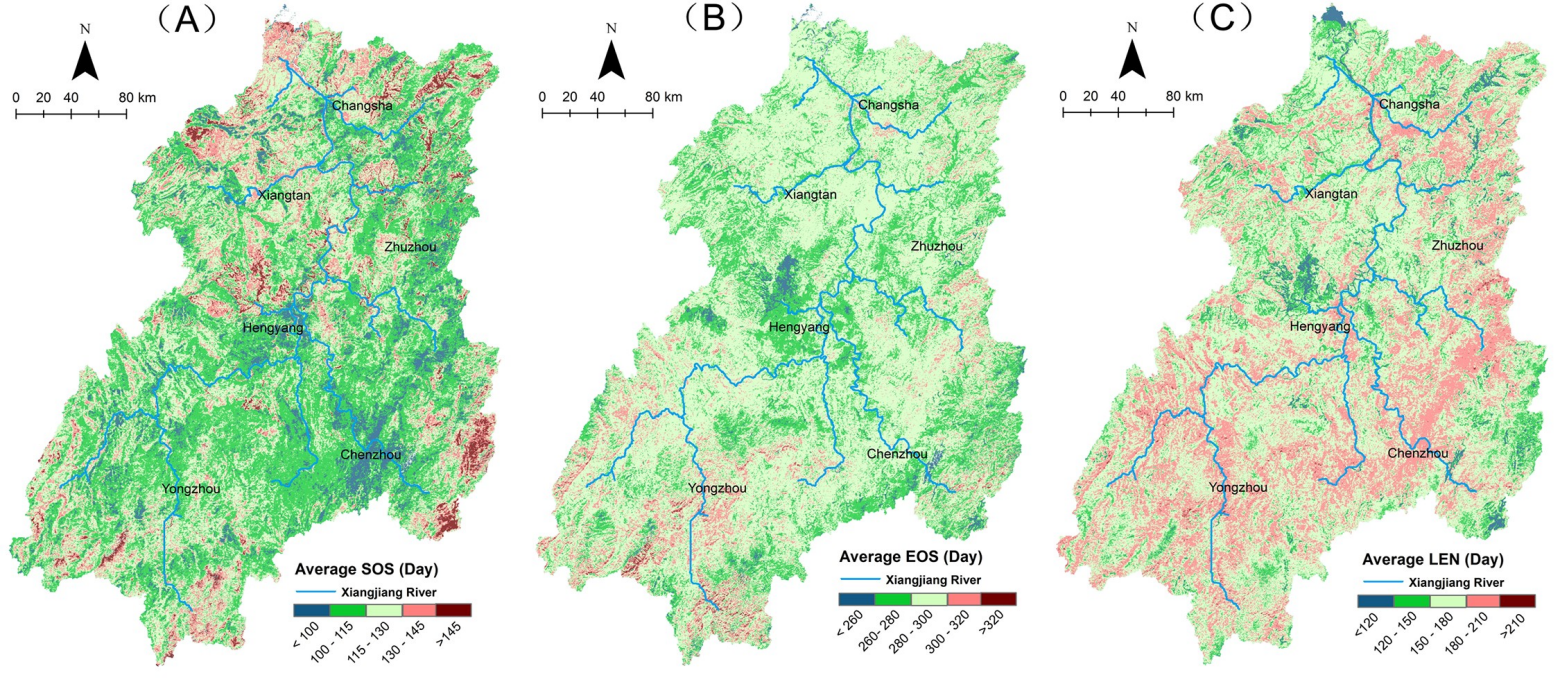
Spatial distribution of phenology in the Xiangjiang River Basin. (A is SOS, B is EOS, and C is LEN. Source of base map was provided by Shuhan Wang. The map was generated by using the free and open source software QGIS version 3.16 (http://www.qgis.org/en/site/)).

Our results show that phenology showed a strong spatial heterogeneity due to the complex topography and hydro-thermal combinations in the Xiangjiang River Basin (shown in Fig 2). Vegetation greened up early in the upper reaches. In the middle and lower reaches, especially in the plain area along the riverbanks, the vegetation had a later SOS and earlier EOS with a shorter growing season.

The roughly same value of the mean and mode shows that the changes of phenology were relatively concentrated in the Xiangjiang River Basin (see Table 1). On average, SOS was at the day of year (DOY) 116.1, mainly ranging between DOY 100–135. The earliest time of green-up was in the first ten days of April (DOY 90–100), and more than 95% of the vegetation greened up by mid-May. The beginning of the whole growing season lasted for one and a half months. The vegetation began to senesce in early September, and the process continued for about two months, ending in late November. The average EOS was at DOY 284.4. However, for about 75% of the area, the EOS was concentrated in October (DOY 270–300). The difference between the beginning and the end of the growing season determines LEN. The average LEN in the basin was 167.8 days, and the difference between the longest and shortest growing season was about 90 days.

**Table 1:** Descriptive statistics of the vegetation phenological indicators.

	Mean	Median	Mode	Standard Deviation
**SOS(day)**	116.1	103.2	108.1	13.63
**EOS(day)**	284.4	272.1	282.4	11.61
**LEN(day)**	167.8	132.2	174.9	19.68

### Interrelation between phenology and annual climatic factors

**Fig 3:**
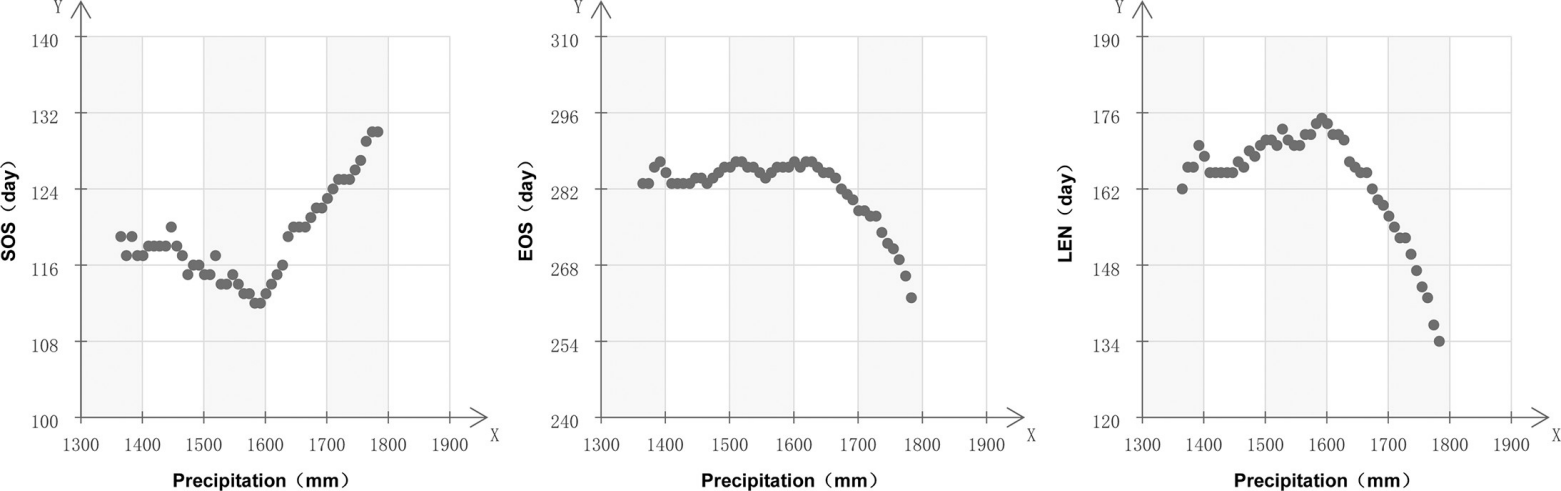
Variation of vegetation phenological indicators as a function of annual precipitation.

The monsoon climate is characterized by a noticeable hot temperature and rainy summer, and cold and wet winter. During 2001–2015, the annual precipitation across the Xiangjiang River Basin varied from 1350 mm to 1750 mm. Fig 3 shows that there was a close relationship between phenology and annual precipitation. Moreover, the change trends of phenology with precipitation showed two distinct intervals. In regions with average yearly precipitation less than 1600 mm, the SOS advanced, the EOS was steady, and the LEN was prolonged with increasing precipitation. In regions with annual precipitation higher than 1600 mm, the correlation coefficients of phenology were more statistically related to precipitation: the SOS was delayed, and the EOS was significantly advanced with the increase of precipitation, resulting in a significant reduction in the LEN.

**Fig 4:**
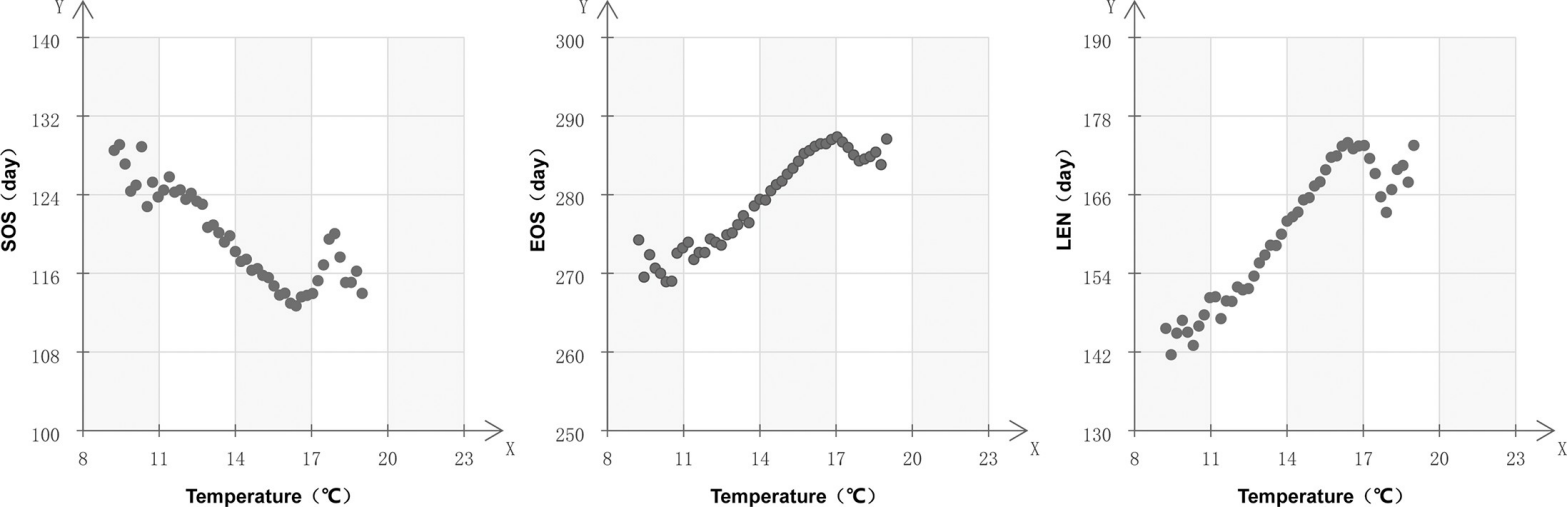
Variation of vegetation phenology as a function of annual mean temperature.

The relationship between phenology and annual temperature was also very close (Fig 4). There were also obvious change intervals. The vegetation growing season significantly lengthened when the temperature was below 16°C, while the SOS advanced and EOS delayed with the increase in temperature. The vegetation phenology fluctuated greatly when the annual average temperature was above 16°C. With the increase in temperature, the SOS was delayed first and then advanced, while LEN was shortened first and then prolonged.

### Vertical changes of phenology

**Fig 5:**
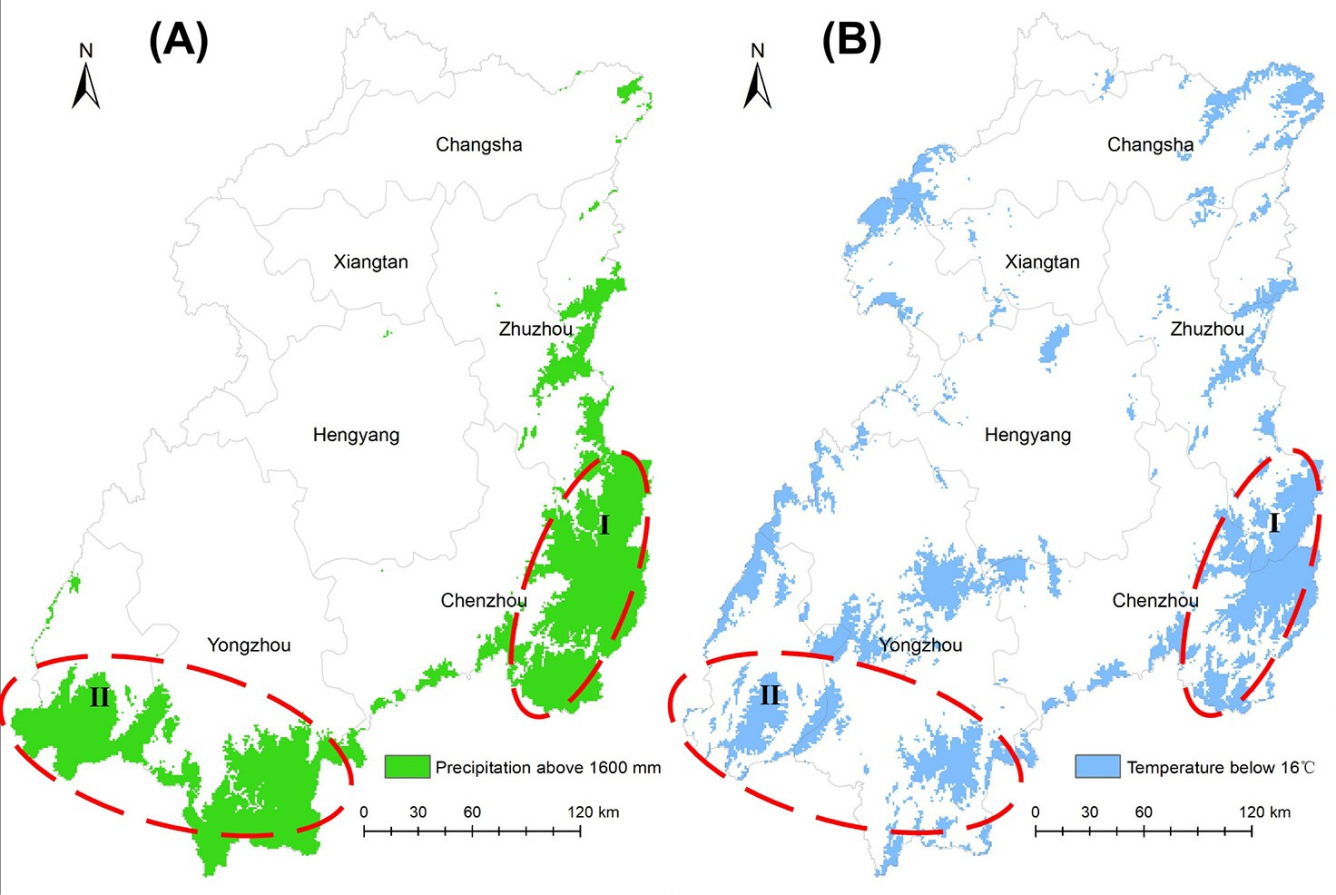
Areas corresponding to phenological turning points. (A shows the areas with annual precipitation greater than 1600 mm; B shows the areas with a yearly average temperature lower than 16°C. The overlapping regions are concentrated in the southeastern part of the upper reaches, and the eastern part of the middle reaches (Luoxiao Mountains, zone I in the figure), and in the southern part of the upper reaches (Nanling Mountains, zone II in the figure). Source of base map was provided by Shuhan Wang. The map was generated by using the free and open source software QGIS version 3.16 (http://www.qgis.org/en/site/)).

As noted above, clear turning points (the temperature of 16°C and the precipitation of 1600 mm) were discovered in the correlation between phenology and yearly temperature/precipitation. We overlaid the areas with annual precipitation greater than 1600 mm and an average annual temperature of less than 16°C during 2000–2015. There were substantial overlaps among these two distribution areas (see Fig 5).

**Fig 6:**
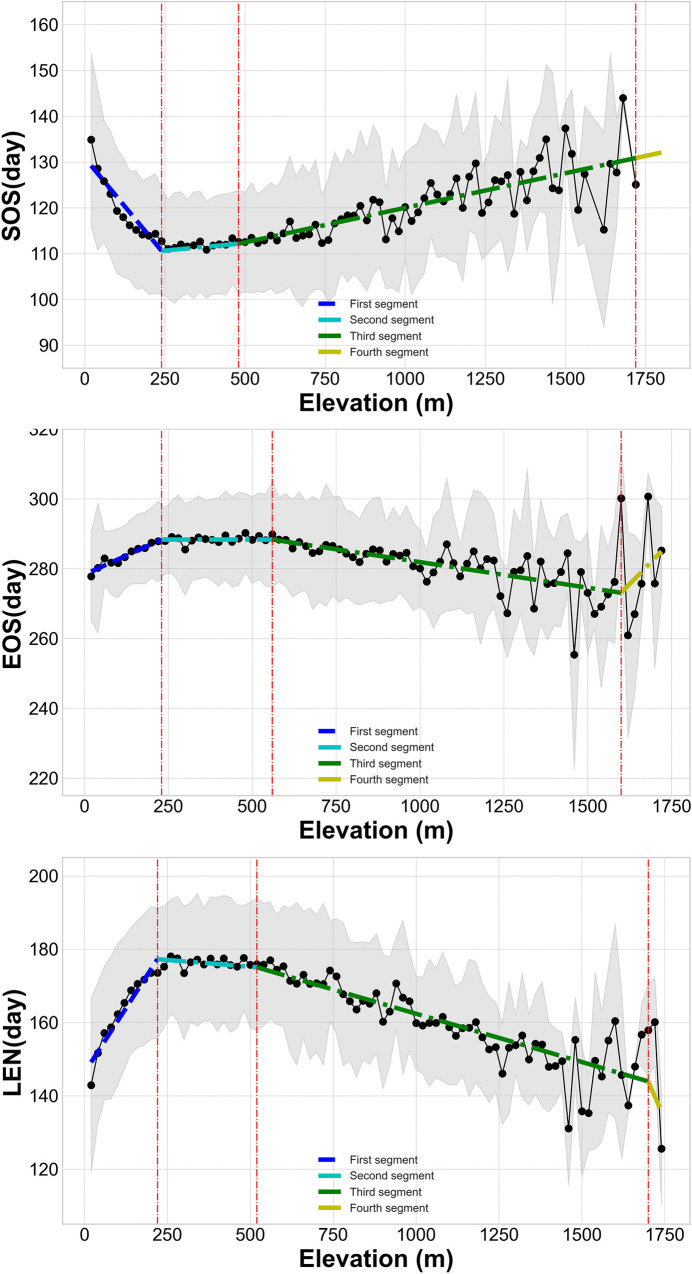
Variation of vegetation phenological indicators as a function of elevation intervals. (In this study, the vertical accuracy of ASTER GDEM V2 is 20m. To analyze the detailed impact of elevation changes on the phenological indicators and also ensure that the data is effective and accurate, the minimum interval of the altitude zoning was set to 20m. The dashed red line represents the turning points of elevation on different phenological indicators; the shaded area shows the standard error of the mean).

To further examine how elevation interacted to produce changes in phenology, we applied a piecewise linear regression [49] to detect the split points and the magnitude of the changes in phenology. As shown in Fig 6, with the change of altitude, the phenology could be divided into four distinct zones. The turning points of SOS were at 240 m, 480 m, and 1800 m; the EOS turning points were at 230 m, 560 m, and 1600 m; at 220 m, 520 m, and 1700 m were the turning points of LEN.

**Fig 7:**
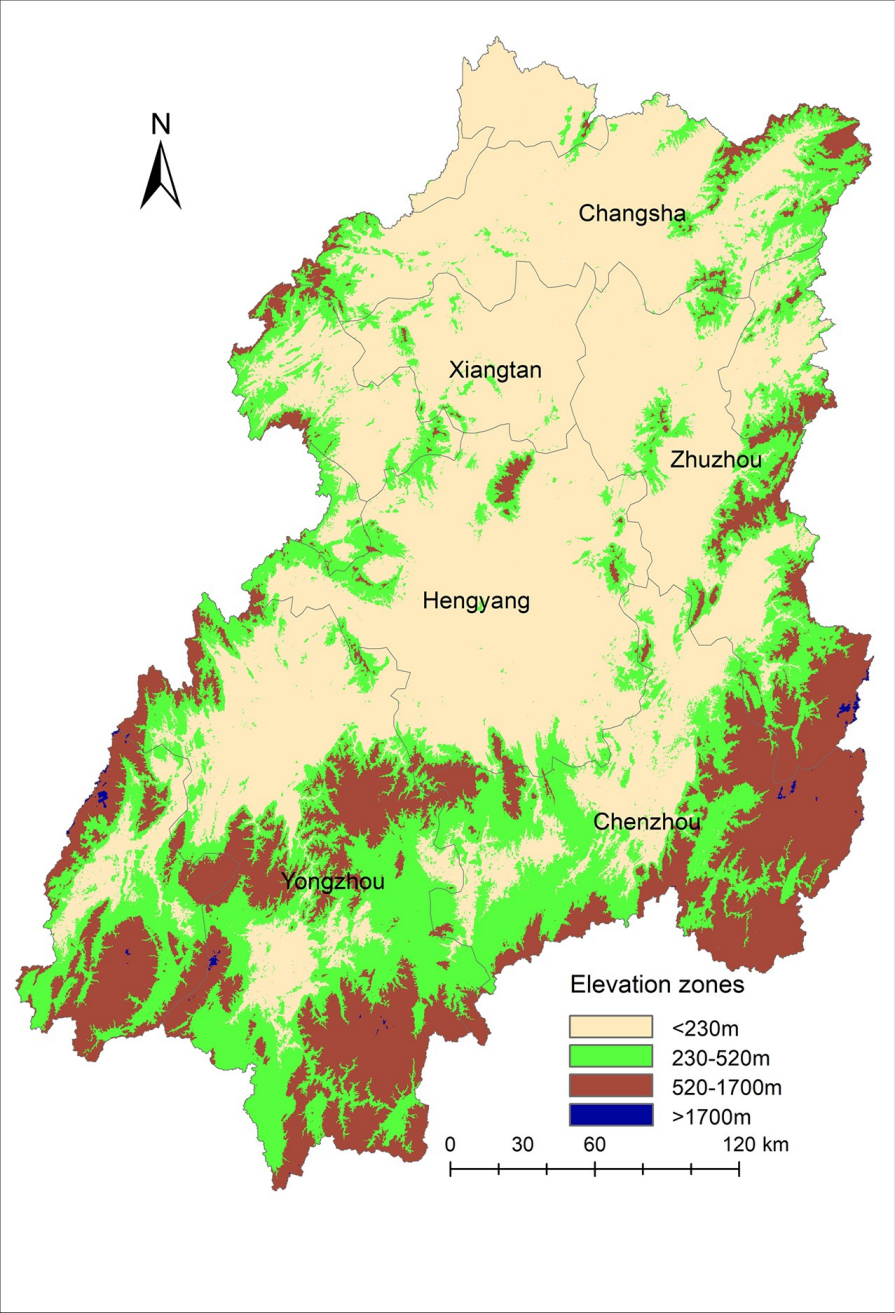
Spatial distribution of elevation zones corresponding to different vegetation phenological areas in the Xiangjiang River Basin. (Numerically, the vegetation phenological zone with an elevation <230 m occupies 53.5% of the area, the zone from 230 to 520 m occupies 27.1% of the area, from 520 to 1700 m 19.3%, and >1700 m accounts for 0.1% of the total basin area. Source of base map was provided by Shuhan Wang. The map was generated by using the free and open source software QGIS version 3.16 (http://www.qgis.org/en/site/)).

The elevation turning points corresponding to phenological indicators were averaged, and 230 m, 520 m, and 1700 m were obtained as the zoning turning points. Specifically, the overlap turning points coincided with the terrain features of the Xiangjiang River Basin. The 230 m and 520 m turning points corresponded to the plain-hills-mountains type elevation feature values (Table 2). The turning point of 1700 m did not correspond to distinct terrain types. As shown in Fig 7, the area above 1700 m was mainly located at the mountain peak. Additionally, this area only accounted for 0.1% of the total basin area. Therefore, to ensure the integrity of the geographical terrain type, we merged the zone above 1700 m into a mountain area. The phenology in the Xiangjiang River Basin was thus divided into three zones: plain vegetation phenology, hilly vegetation phenology, and mountain vegetation phenology.

**Table 2:** Relationship between the geographies of five basic terrain types and elevation features.

Terrain type	Elevation feature description*
**Plain**	Elevation below 200m
**Hilly**	Elevation ranges from 200m to 500m, and the relative height does not exceed 200m
**Mountain**	Elevation above 500m
**Plateau**	Elevation above 1000m, the terrain is relatively flat
**Basin**	No specific elevation or relative height feature; the only surrounding terrain is higher than the middle

(*The definition and elevation feature of the five basic terrain types referenced from the Chinese geomorphology encyclopedia.)

### Phenology in the three identified zones

**Fig 8:**
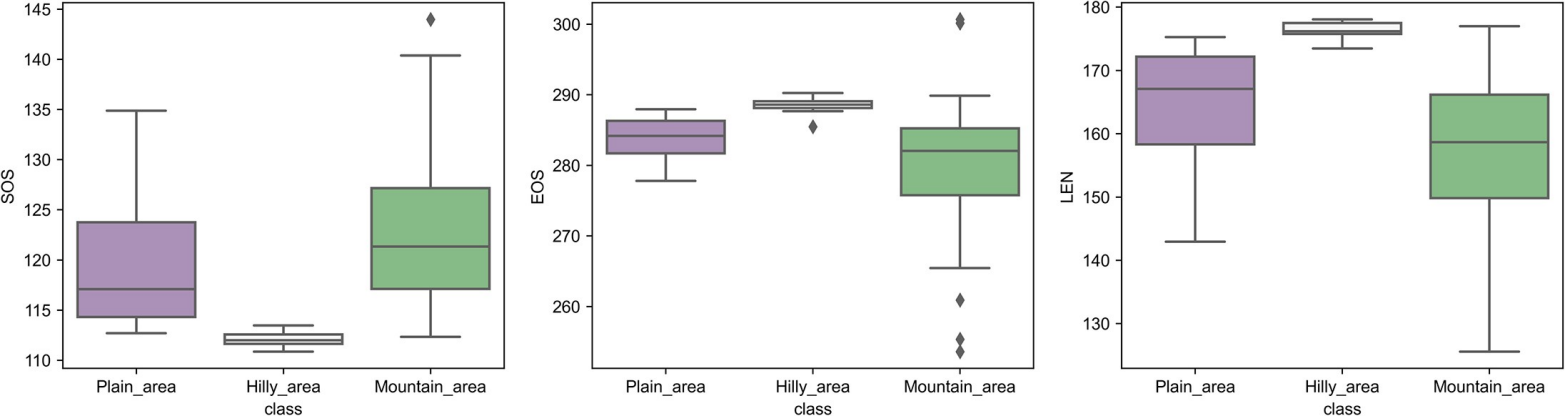
Box plots of different phenological indicators in the three identified vegetation phenological areas.

The SOS, EOS, and LEN presented unique distribution patterns in the three identified phenological zones (Fig 8). Compared with the other two zone areas, the hilly zone area was characterized by the lowest interquartile range, suggesting that vegetation in the hilly areas had a concentrated growth period, and SOS, EOS, and LEN were all in concentrated periods. In contrast, the maximum and minimum values in the mountain area had the largest separation and the longest-range value, indicating that the phenological changes were drastic fluctuation in the mountains. Among the three identified zones, the median value of SOS in the plain area deviates from the maximum value. That is, a lot of vegetation phenology might have changed rapidly in a certain period.

On the other hand, the linear fitting equations slope (shown in Table 3) indicated that for every 100 m of rising altitude, SOS was advanced by 7–8 days, EOS was delayed by 5–6 days, and LEN increased by 14–15 days in the plain area. However, in the hilly area, the r-squared of the linear fitting equations was low, and all fitting equations did not pass the significance test. In other words, there were no clear relationships between phenology and elevation. At an elevation greater than 520 m, the mountainous area was another significant area where phenology changed with elevation. The linear fitting equations showed that the r-squared between phenology and elevation was as high as 0.95, and the slope of the linear fitting equations demonstrated that as the altitude increased by 100m, SOS was delayed by 1–2 days, EOS was advanced by 1–2 days, and LEN was shortened by 2–3 days.

**Table 3:** The linear fitting results and significance test between phenology and elevation.

	Elevation	Fitting equation parameters			
		Y-intercept	Slope	r-squared	F-value
**SOS**	ALL	110.326	0.011	0.688 (p<0.001)	218.86
	<240m	129.333	-0.077	0.888 (p<0.001)	88.59
	240-480m	112.956	-0.003	0.109 (p = 0.144)	2.47
	480-1800m	104.260	0.0151	0.984 (p<0.001)	3913.87
	>1800m	54.455	0.043	0.642 (p = 0.002)	18.92
**EOS**	ALL	288.282	-0.008	0.584 (p<0.001)	139.71
	<230m	276.673	0.053	0.919 (p<0.001)	113.82
	230-560m	286.422	0.005	0.257 (p = 0.022)	6.53
	560-1600m	296.980	-0.016	0.978 (p<0.001)	2260.57
	>1600m	227.472	0.026	0.539 (p<0.001)	24.35
**LEN**	ALL	177.750	-0.018	0.699 (p<0.001)	230.38
	<220m	144.751	0.149	0.920 (p<0.001)	115.40
	220-520m	173.750	0.888	0.343 (p = 0.01)	8.81
	520-1700m	191.229	-0.029	0.992 (p<0.001)	7248.20
	>1700m	140.112	0.001	0.003 (p = 0.843)	0.04

#### Relationship between phenology and influencing factors in three identified zones

**Fig 9:**
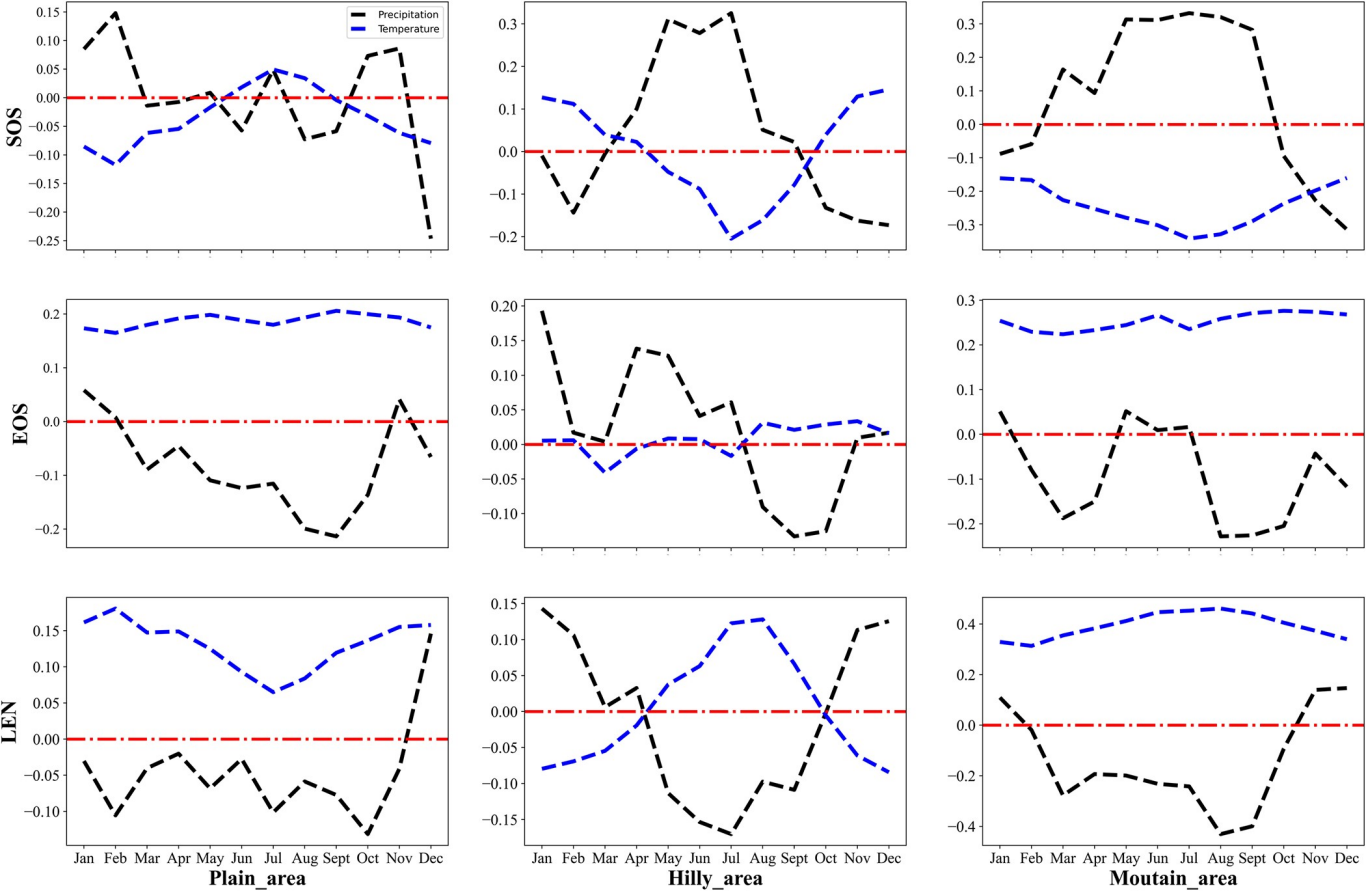
The relationship of the different phenological indicator values and monthly temperature/precipitation in the three identified vegetation phenological areas.

As shown in Fig 9, the Spearman correlation coefficients suggest that the correlations between the phenology and monthly temperature/precipitation are significantly different depending on the region. In the hilly and mountain areas, the SOS showed a positive correlation with precipitation from May to July and showed a negative correlation with July temperature. However, the correlation between EOS and temperature in the plain and mountain areas showed that there was a positive correlation with temperature for the entire year. The correlation between LEN and temperature/precipitation in the plain and mountainous areas was similar to the change of EOS with temperature/precipitation. In the hilly area, the correlation between LEN and temperature/precipitation shows opposite characteristics. Specifically, LEN was negatively correlated with summer precipitation or winter temperature and positively correlated with winter precipitation or summer temperature.

The temperature, precipitation, elevation, vegetation types, and phenological indicators were imported into the geographic detector model to calculate the spatial influence values of the three identified zones (Table 4). The primary factors (where q > 0.1) affecting SOS were precipitation (0.1632), temperature (0.1421), and vegetation types (0.1232) in the plain area. However, all factors had weak effects on EOS. By contrast, in the mountain area, the largest factor affecting SOS was precipitation (0.2212), and the primary factors affecting EOS were temperature (0.2032) and elevation (0.1159) (P-value of elevation on EOS was not significant). Unlike plain and mountain areas, in the hilly area, only vegetation types had a significant impact on SOS (but the P values did not achieve significance thresholds). Overall, precipitation was the most crucial factor that affected SOS both in plain and mountain areas. There was no significant factor that affected EOS in the plain area, but temperature had an essential effect on EOS in the mountain area.

**Table 4:** The contribution of factors spatially influencing phenology in the three identified zones.

	Influencing factors	Plain area		Hilly area		Mountain area	
		q statistic	p-value	q statistic	p-value	q statistic	p-value
**SOS**	Elevation	0.0013	1.0000	0.0588	0.0000	0.0856	1.0000
	Precipitation	0.1632	0.0000	0.0767	1.0000	0.2212	0.0000
	Temperature	0.1421	0.0000	0.0692	1.0000	0.0499	1.0000
	Vegetation type*	0.1232	0.0000	0.1203	0.9878	0.0116	1.0000
**EOS**	Elevation	0.0241	1.0000	0.0196	1.0000	0.1160	1.0000
	Precipitation	0.0232	1.0000	0.0812	1.0000	0.0303	1.0000
	Temperature	0.0246	1.0000	0.0099	1.0000	0.2032	0.1000
	Vegetation type*	0.0992	1.0000	0.0745	1.0000	0.0076	1.0000
**LEN**	Elevation	0.0118	0.6211	0.0685	0.0000	0.2714	0.0000
	Precipitation	0.0405	0.0000	0.0246	1.0000	0.1052	0.6880
	Temperature	0.0527	0.0000	0.0204	1.0000	0.3328	0.0000
	Vegetation type*	0.1537	0.0000	0.1557	0.7473	0.0111	1.0000

(*The vegetation types in different elevation intervals zones were obtained from the land use data of 2015 in the Xiangjiang River Basin. The data wasdownloaded from the FROM-GLC website(http://data.ess.tsinghua.edu.cn/).)

## Discussion

### Vegetation distribution pattern and phenology

Numerous studies have reported gradual longitudinal or latitudinal zonal changes of phenology at the national or intercontinental scale, especially in areas that are very sensitive to climate change [8, 13, 32, 36, 50]. It is generally believed that vegetation activity at large scales is affected by temperature variability and water stress [26, 28, 32, 37, 51, 52]. In this study, our results show that the spatial distribution characteristics of phenology are very heterogeneous at a watershed scale, and the three phenological indicators do not gradually change longitudinally or latitudinally in the Xiangjiang River Basin. For climatic factors that interactively act on phenology, topographical features, and vegetation types may have a more important influence in this region area. According to a previous investigation [53], natural and cultivated vegetation types are staggered in the Xiangjiang River. There are a large number of broad-leaved forests, coniferous forests, and pine forests distributed in the mountains area. In contrast, crops extend in the middle and lower reach. Therefore, the vegetation types are fragmented and result in an intricate distribution of phenology.

Specifically, we have observed that phenology presents a striped variation similar to the river network of the Xiangjiang River. Earlier SOS (mid-April) mainly appears in the hilly or low mountainous areas, which are far away from the river. In those areas, broad-leaved forests are the main vegetation type. Most of the broad-leaved woodlands begin to green up their leaves in early and mid-April. These dates are consistent with the SOS of subtropical forests in the middle and lower reaches of the Yangtze River under the same climate belt [17]. In contrast, later SOS appears in a large area of the midstream and downstream reaches. As one of the most important food production areas in southern China, there are large croplands along the Xiangjiang River bank, and the "early rice-late rice" [24] are the mainly rice planting modes in these areas. Due to the higher daily accumulated temperature required for the cultivation of rice, the planting time is basically in the middle of May; therefore, it leads to the emergence of a late SOS.

### Vertical zoning characteristics of phenology

It also has been recognized that the elevation significantly contributed to the variability in phenology [28, 39, 54]. Usually, these vertical changes occur in the mountains or plateaus with great fluctuations in altitude, such as in the Alps [31, 56] or the Qinghai-Tibet Plateau [54–57]. Our analysis highlighted that three distinct phenological zones could be explored using the elevation thresholds of less 230 m, 230 to 520 m, and above 520 m in the Xiangjiang River Basin. This phenomenon is not unique. In the Danjiangkou watershed (located north of the Xiangjiang River Basin), elevation-dependent precipitation/temperature differences have also caused a transition in phenological change [34]. In the Qilian Mountains, the correlation between green-up and precipitation gradually weakened over the altitudinal gradient [28]. These results are not contradictory but reflect the following reality: In general, altitude change is the primary influencing factor in changing temperature [39, 58]. As altitude increases, the decrease in temperature can inhibit the growth of vegetation and lengthen the time for vegetation to reach the accumulated growth temperature [59]. However, the changes in elevation-dependent precipitation do not always present the same trend [60]. In complex topography, precipitation may undergo complex changes with terrain, and its direct influence on soil moisture, especially winter precipitation, improves the effective soil water content which is closely linked to the green-up of vegetation [52, 61].

It is clear that phenology can be strongly controlled by temperature increasing or precipitation changing, and it may shift in unexpected ways in the climate-sensitive area [20, 26, 28]. Nevertheless, the shift of turning points with climate change was inconspicuous in the Xiangjiang River Basin, as the multi-annual average temperature and precipitation only slightly oscillated during our study period. According to our results, some general conjectures could be proposed. In the three identified zones, the responses of phenology to climate change might be divergent. And climate change with increasing temperature variation might cause raised SOS turning points, and decreased EOS turning points. These conjectures, however, still need future work to verify by collecting more phenological data.

### Factors influencing phenological change

In most high-altitude terrestrial ecosystems, vegetation phenological shifts have been unequivocally attributed to climate change, particularly to temperature [3, 8, 14, 20, 32, 62]. As expected, our correlation analysis and geographic detectors results show that temperature was the primary factor affecting the LEN in the Xiangjiang River Basin, but this impact was quite different in three vertical zoning areas. For the explanatory factors, temperature had a significant impact on mountainous LEN; as the temperature increases, LEN increases significantly. Our results suggest that temperature changes to LEN were mainly directly related to the postponement of EOS through temperature increase because the geographic detector results showed temperature was not the main factor affecting SOS in mountainous areas. As many previous studies have affirmed, warmer autumn leads to a later EOS [17, 55]. Usually, in mountainous areas, where the temperature is low, the increase of effective accumulated temperature will have a significant impact on vegetation growth [33]. In particular, a higher temperature in autumn can ensure that the vegetation has greater energy accumulation, which will have an important effect on enhancing the activities of photosynthetic enzymes and delaying the senescence of vegetation [63].

Precipitation is another major factor affecting phenology [26, 28, 39, 52]. Our correlation analysis shows that there were large distinctions between precipitation and phenology in different months. The geographic detectors results emphasize that precipitation had a significant effect on SOS in plain and mountain areas. Furthermore, the correlation analysis results show there was a positive correlation between spring precipitation and SOS. A similar precipitation effect was also observed in the evergreen broad-leaved forest of southern China [17]. This is mainly due to an increase in spring precipitation in the mountains, which not only increases soil moisture but also reduces temperature and indirectly reduces soil evaporation so that soil moisture can reach its optimal state earlier, and vegetation can begin to grow earlier [26]. However, we also noticed that the correlation between winter precipitation and SOS was higher, but there was a negative correlation between the two. One possible reason for the deferred SOS is related to reduced absorption of solar radiation, which is proportional to the amount of winter precipitation [16, 52]. Generally, precipitation is closely correlated with cloudy days. Increases in cloudy days following larger winter precipitation might lead to a decrease in the chilling accumulation and insolation, which could delay SOS in forests [64].

Although this study mainly analyzed the critical climatic factors that control vegetation growth, several other external factors have a profound impact on vegetation phenology, such as human disturbance, species competition, and pests [7]. Among these factors, human disturbance, especially land-use change caused by rapid urbanization, is considered one of the main reasons leading to the changes in vegetation phenology [65]. A previous study [53] has shown that forest land and farmland are the two major vegetation types in the Xiangjiang River Basin. Particularly, in the plain area, farmland is the major type and accounted for 38% of the distribution area at the time of our study. More importantly, nearly 82% of the farmland in the entire basin is distributed in this area. As a consequence, the geographic detectors show that vegetation type was also the main factor affecting SOS in the plain areas. This is mainly because the planting and harvesting period of farmland introduces an artificially later SOS and earlier EOS [24].

It is worth mentioning that the implementation of the shelter forest project has increased the forest coverage from about 45% in 1994 to about 60% in 2018 in the Xiangjiang River Basin. Therefore, in future work, the quantitative impact of land change on phenology in different vegetation phenological zones will need to be clarified.

## Conclusions

Our study area, the Xiangjiang River Basin, is located in the center of the subtropical monsoon climate region of China. The spatial pattern of phenology is closely related to both climate and topographical factors. Temporally, the average SOS began to shift to the period DOY 100–135, and the average EOS was between DOY 270–330. Spatially, phenology presented a striped variation similar to the river network. We have shown that elevation played an important role in the differentiation of phenology at a watershed scale. Using elevations of 320 m and 520 m as the boundary, we identified three distinct vegetated areas. The primary factors affecting phenology were quite different in the three vertical zoning areas. Precipitation was the most crucial factor affecting SOS both in plain and mountain areas. Temperature had an essential effect on EOS in the mountain area. These results indicate the importance of elevation in the study of regional vegetation phenology and our understanding of the subtropical monsoon ecosystem responses to climate change.

## Supporting information

S1 Data Analysis data. (RAR)

S2 Data Phenology data. (RAR)
